# CB1 receptor antagonist rimonabant protects against chronic intermittent hypoxia-induced renal injury in rats

**DOI:** 10.1186/s12882-021-02362-6

**Published:** 2021-04-26

**Authors:** Li Zhao, Tao Liu, Zhan-jun Dou, Mei-ting Wang, Zi-xuan Hu, Bei Wang

**Affiliations:** 1 Shanxi Medical University, No. 56, Xijian South Road, Taiyuan, 030001 Shanxi People’s Republic of China; 2 The Second Hospital of Shanxi Medical University, No. 382, Wuyi Road, Taiyuan, 030001 Shanxi Province People’s Republic of China

**Keywords:** Obstructive sleep apnoea, Chronic intermittent hypoxia, Cannabinoid receptor system 1;renal injury; mitochondrial dynamics, Rimonabant

## Abstract

**Background:**

Obstructive sleep apnoea (OSA) induced chronic kidney disease is mainly caused by chronic intermittent hypoxia (CIH). Our study investigate the mechanism underlying CIH-induced renal damage and whether the cannabinoid receptor 1 (CB1R) antagonist rimonabant (Ri) alleviates CIH-induced renal injury.

**Methods:**

Male Sprague-Dawley rats were randomly divided into five groups: one normal control (NC) group, two chronic intermittent hypoxia (CIH) groups, and two CIH + Ri groups. Rats in the NC groups were exposed to room air, while the CIH groups were exposed to a CIH environment for 4 weeks (4w CIH group) and 6 weeks (6w CIH group), respectively. Additionally, rats in the CIH + Ri groups were administered 1.5 mg/kg/day Ri for 4 weeks (4w CIH + Ri group) and 6 weeks (6w CIH + Ri group), respectively. Following this, the rats were euthanized and kidneys were excised for downstream analysis. In the renal tissues, the morphological alterations were examined via haematoxylin eosin (HE) staining and periodic acid schiff (PAS) staining, CB1R, Fis1, Mfn1, and p66Shc expression was assessed through western blot and immunohistochemistry, and the mitochondrial ultrastructural changes in kidney sections were assessed by electron microscopy.

**Results:**

CB1R expression in the 4w and 6w CIH groups was significantly elevated, and further increased with prolonged hypoxia; however, Ri prevented the increase in CIH-induced CB1R expression. Fis1 and p66Shc expression in the CIH groups were increased, but Mfn1 expression decreased. Ri decreased Fis1 and p66Shc expression and increased Mfn1 expression. Renal damage in the 4w or 6w CIH + Ri group was evidently improved compared with that in the 4w or 6w CIH group. CB1R expression was positively correlated with Fis1 and p66Shc and negatively correlated with Mfn1. Meanwhile, electron microscopy showed that the percentage of fragmented mitochondria in the tubular cells in each group was consistent with the trend of CB1R expression.

**Conclusion:**

CIH causes endocannabinoid disorders and induces abnormal mitochondrial dynamics, resulting in renal injury. Treatment with CB1R antagonists reduces CIH-induced renal damage by inhibiting dysregulated renal mitochondrial dynamics.

**Supplementary Information:**

The online version contains supplementary material available at 10.1186/s12882-021-02362-6.

## Background

Obstructive sleep apnoea (OSA) is one of the most common sleep respiratory disorders that may present with or without symptoms. Approximately close to one billion people are affected, with the prevalence exceeding 50% in some countries [1]. The pathogenesis of OSA is characterised by repeated upper airway obstruction, which causes partial or whole upper airway closure leading to apnoea and arousal. Chronic intermittent hypoxia (CIH) is a central dominant feature of OSA [2], and produces serious damage, which resembles that caused by ischaemia-reperfusion injury [3]. Eventually, CIH is recognized as an independent risk factor for multiple diseases, including coronary heart disease, hypertension, pulmonary heart disease, pulmonary embolism, Alzheimer’s disease, Parkinson’s syndrome and stroke [4–9]. Increasing evidence from the past two decades indicates that patients with untreated OSA have increased risk of advanced chronic kidney disease (CKD) [10]. Recent studies revealed that OSA contributes to CKD via intrarenal hypoxia [11]. Developing a new therapeutic approach for OSA-induced CKD can have significant implications for clinical practice and reduce the population health burden [12].

Recent studies showed that CIH could cause target organ damage by regulating mitochondrial function [13, 14]. Tubular epithelial cells have enriched mitochondria due to high energy demand. Thus, the relevance of mitochondria in the pathogenesis of kidney disease has been extensively investigated [15]. Previous studies indicate that mitochondrial impairment and mitochondrial-derived oxidative stress substantially contribute to tubular cell injury and induces apoptosis in kidney disease [16, 17]. Mitochondria are dynamic organelles and are a major source reactive oxygen species (ROS) production. Excessive ROS leads to oxidative stress and mitochondrial dysfunction, leading to cell ageing, injury, and apoptosis [18]. Mitochondria constantly undergo fission and fusion to maintain a healthy mitochondrial pool. These mitochondrial dynamics are finely regulated by the pro-fusion mitofusins, Mfn and OPA1, and the pro-fission proteins, Drp1 and Fis1. Excessive fusion or division inhibits sufficient mitochondrial ATP production, leading to cell damage and apoptosis [19]. Mitochondrial fragmentation is a morphological change and an early critical process contributing to mitochondrial membrane leakage and consequent cell death [16]. P66Shc is an adaptor protein belonging to the ShcA family, and may modulate mitochondrial pathobiology in the kidney [20, 21]. Some studies show that p66Shc activation and phosphorylation induces mitochondrial fragmentation, increases interactions between fission proteins and apoptogenic factors, and activates downstream apoptotic pathways [22].

Identifying molecules in CIH that modulate mitochondrial dynamics and cause functional disturbances in kidney disease is imperative for the implementation of therapeutic strategies. Recent studies provide new insights into cannabinoid receptor 1 (CB1R), which affects mitochondrial function through the regulation of mitochondrial dynamics in the kidney. Further, CB1R activation leads to excessive mitochondrial fracture, and CB1R knockdown in mouse proximal tubule cells have reduced mitochondrial fragmentation and dysfunction [23]. CB1R is a G-protein coupled receptor, and is part of the endocannabinoid (EC) system [24]. EC signalling regulates various physiological functions in vivo [25]. CB1R is expressed in several tissues such as adipose tissue, liver, skeletal muscle, and kidney. In renal tissue, CB1R is localised in podocytes, mesangial cells, proximal tubules, and distal tubules [26]. Previous studies suggest that CB1R is upregulated in human kidney disease, leading to renal hemodynamic abnormalities and dysfunction, oxidative stress, inflammation, and renal fibrosis, which play an important role in kidney disease [27–29]. Currently, CB1R antagonists have been used to inhibit CB1R overexpression in animal experiments, but whether these antagonists can alleviate or prevent CIH-mediated renal injury is unclear.

We hypothesised that OSA may cause target organ damage via abnormal regulation of mitochondrial dynamics, which may be regulated by the ECs. Therefore, we established an OSA-CIH rat model and investigated how CIH influences renal tissue mitochondrial dynamics. We examined p66Shc, Fis1, and Mfn1 expression in renal tissue to assess the mitochondrial dynamics. Next, we investigated the changes in CB1R expression in our OSA-CIH model after the administration of rimonabant (Ri), a CB1R antagonist. Finally, we compared kidney damage and the expression of mitochondrial dynamics factors between the CIH + Ri and CIH groups to determine whether CB1R could be a therapeutic target that could relieve or prevent CIH-mediated renal injury.

## Methods

### Experimental animals

Forty healthy male Sprague-Dawley rats (450–500 g, 8–10 weeks old) were purchased from the Shanxi Medical University Animal Center, China. The rats were housed under standard conditions at 22–24 °C and a 12 h light/dark cycle. Animal care was in compliance with the legal requirements and guidelines approved by the Ethics Committee for Animal Facility of Shanxi Medical University. All efforts were made to minimise animal suffering.

The rats were randomly divided into five groups with eight rats each: one normal control (NC) group, two chronic intermittent hypoxia (CIH) groups, and two CIH + Ri groups. Rats in the NC group breathed room air. Rats in the CIH groups experienced intermittent hypoxia (see below) for 4 (4w CIH group) or 6 weeks (6w CIH group). The rats in the CIH + Ri groups were given a daily intraperitoneal injection of 1.5 mg/kg Ri for 4 (4w CIH + Ri group) or 6 weeks (6w CIH + Ri group).

### CIH model

A hypoxic control animal experiment system A84 (BioSpherix, Parish, NY, USA) was used to produce the hypoxic environment. A gas control system was used to regulate oxygen and nitrogen flow into the chamber. Ambient oxygen was servo-controlled to generate intermittent hypoxia. During a 2-min cycle, nitrogen was pumped into the chamber at a fixed rate to reach 8% fraction of inspired oxygen (FiO_2_) within 30 s. Then, compressed air was introduced into the chamber at 10 L/min to achieve 21% FiO_2_ within 50 s. Compressed air flow was reduced to 5 L/min to maintain the level of 21% oxygen for the remaining 40 s per cycle. Rats were placed into the chamber for 30 cycles per h, 8 h per day, for four or six consecutive weeks. Rats in the NC group were housed in the chamber with 21% FiO_2_ for the entire experiment. Rats in the CIH + Ri groups received a daily intraperitoneal Ri injection prior to placement into the chronic intermittent low-oxygen chamber. The oxygen concentration in the chambers was verified using a portable oxygen analyser, and an electrode was inserted into the chamber to confirm all oxygen content changes [30].

### Haematoxylin and eosin staining

After 4 or 6 weeks, the renal tissues of the rats were collected and placed in 4% paraformaldehyde for 24 h. The fixed renal tissues were dehydrated with gradient alcohol, embedded in paraffin, and made into 4 μm thick pathological sections. The dried slices were immersed in xylene I/II/III in sequence for 10 min each, then absolute ethanol, 95, 80, 70% ethanol for 7 min each, and washed with water for 5 min. The slices were stained in haematoxylin for 5 min and washed with water for 5 min, differentiated by 1% hydrochloric acid in alcohol for 5 s and rinsed with water for 1 min, counterstained with 1% eosin for 10 s and then washed for 1 min. They were immersed in 70, 80, 95% and absolute ethanol each for 2 min, then immersed in xylene I/II for 5 min each. Finally, once the sections were naturally dried, they were sealed with neutral gum, and imaged with a light microscope (Olympus, Tokyo, Japan).

### Periodic acid schiff staining

Firstly, the tissue slices were routinely dewaxed to water, oxidized with 10% periodic acid solution for 10 min, cleaned with distilled water, then put into Schiff dye solution for 15 min, washed with running water for 5 min, stained with hematoxylin for 3 min, washed with running water for 5 min, and then dehydrated, transparent and sealed with resin.

Using a previously reported scoring system [31], we assigned histopathological scores. Histological changes due to tubular necrosis were quantitated by calculating the percentage of tubules in cell necrosis, loss of brush border, cast formation, and tubule dilatation as follows: 0, none; 1, ≤10%; 2, 11–25%; 3, 26–45%; 4, 46–75%; and 5, > 76%. At least 10 fields (× 200) were reviewed for each slide.

### Electron microscopy

Kidney tissues (1 mm3) were cuted by a sharp blade quickly within 1–3 min processed for further fixation. And then wash the tissues using 0.1 M PB (pH 7.4) for 3 times, 15 min each. Tissues avoid light post fixed with 1% OsO4 in 0.1 M PB (pH 7.4) for 2 h at room temperature, and rinsed in 0.1 M PB (pH 7.4) for 3 times, 15 min each. The tissues were successively dehydrated with gradient alcohol for 20 min each time, 100% acetone twice, 15 min each time. Then, resin penetration and embedding at 37 °C, insert the tissues into the pure EMBed 812, and keep in 37 °C oven overnight. The embedding models were moved into 65 °C oven to polymerize for more than 48 h, and were cut to 60-80 nm thin on the ultra microtome, then the tissues were fished out onto the 150 meshes cuprum grids with formvar film. Then 2% uranium acetate saturated alcohol solution avoid light staining for 8 min, rinsed in 70% ethanol for 3 times and then rinsed in ultra pure water for 3 times. 2.6% Lead citrate avoid CO_2_ staining for 8 min, and then rinsed with ultra pure water for 3 times. After dried by the filer paper, the cuprum grids were put into the grids board and dried overnight at room temperature. At last, the cuprum grids are observed under TEM 120 kv (HITACHI, Tokyo, Japan) and take images.

To determine mitochondrial fragmentation, the length of mitochondria in 8 randomly selected tubular cells from each group (> 100 mitochondria per cell) were measured. The mitochondrion having length > 2 μm were considered filamentous and those with < 1 μm and spherical configuration were designated as fragmented [21].

### Immunohistochemistry

The renal sections were dried in an incubator at 65 °C for 20 min, deparaffinised with xylene I/II/III for 10 min each, rehydrated in absolute ethanol, 95, 80, 70% ethanol each for 7 min, and immersed in water for 5 min each. Then, sections were placed in EDTA (pH 8.0) and heated in a microwave for 2.5 min for antigen retrieval. Three percent hydrogen peroxide solution was evenly dropped onto the tissue section, covered with a wet lid, and treated with dark treatment for 15 min to inactivate endogenous peroxidase. Samples were washed twice in PBS for 5 min and blocked with 10% normal goat serum in PBS at 25 ± 1 °C for 30 min. The sections were stained overnight at 4 °C with rabbit anti-rat anti-CB1R (1:100), anti-Mfn1 (1:100), anti-Fis1 (1:100), and anti-p66Shc (1:50; Santa Cruz Biotechnology, Dallas, TX, USA). Then, the sections were incubated with biotinylated goat anti-rabbit secondary antibody (1:100, Santa Cruz Biotechnology), for 40 min at 37 °C. Diaminobenzidine (DAB) was used to develop the colour for 5 min, and the reaction was quenched with distilled water. Then, the sections were incubated in haematoxylin staining solution for 15 s, rinsed with water for 5 min, and incubated acid alcohol. Finally, tissue samples were dehydrated with graded ethanol (95, 100%) for 2 min each, cleared with xylene I/II three times for 2 min each, and mounted. Samples were imaged with a scanscope CS2 digital pathological scanning system (Leica Biosystems, Wetzlar, Germany) to analyse CB1R, p66Shc, Fis1, and Mfn1 expression in renal tissues.

### Western blotting analysis

Whole kidneys were lysed in lysis buffer and centrifuged at 14000 *g* for 20 min at 4 °C. The supernatant was then collected. Nuclear and cytosolic renal proteins were extracted using a protein extraction kit (Invitrogen, Carlsbad, CA, USA) according to manufacturer’s instructions. Protein concentration was determined using a BCA protein assay kit. Total protein (50 μg) was separated during 12% SDS polyacrylamide gel electrophoresis. Separated proteins were transferred to polyvinylidene difluoride membranes (Millipore, Burlington, USA) using a semi-dry transfer blotting apparatus. The membranes were incubated with primary antibodies for 18 h at 4 °C. The primary antibodies included rabbit anti-rat anti-CB1R (1:500) anti-p66shc (1:500), anti-Fis1 (1:500), anti-Mfn1 (1:500; Santa Cruz Biotechnology). After washing with TBST, the membranes were incubated with the horseradish peroxidase-conjugated secondary antibody goat anti-rabbit (1:5000 Santa Cruz Biotechnology, USA) for 60 min at 25 ± 1 °C. Add ECL chemiluminescence reagent, reaction 1 min at room temperature, X-ray film exposure development, fixing. Protein expression was measured with Image J (National Institutes of Health, Bethesda, MD, USA). Relative target protein expression was normalised to β-actin.

### Statistical analysis

Statistical analyses were performed using SPSS 19.0 software (IBM, Armonk, NY, USA). The data were expressed as mean ± SEM. Statistical differences were evaluated by one-way analysis of variance (ANOVA). Correlation analyses were carried out using Pearson’s correlation and Spearman’s correlation analysis. Data with *P* < 0.05 were considered statistically significant.

## Results

### Pathological changes in the kidney from CIH

**Fig. 1:**
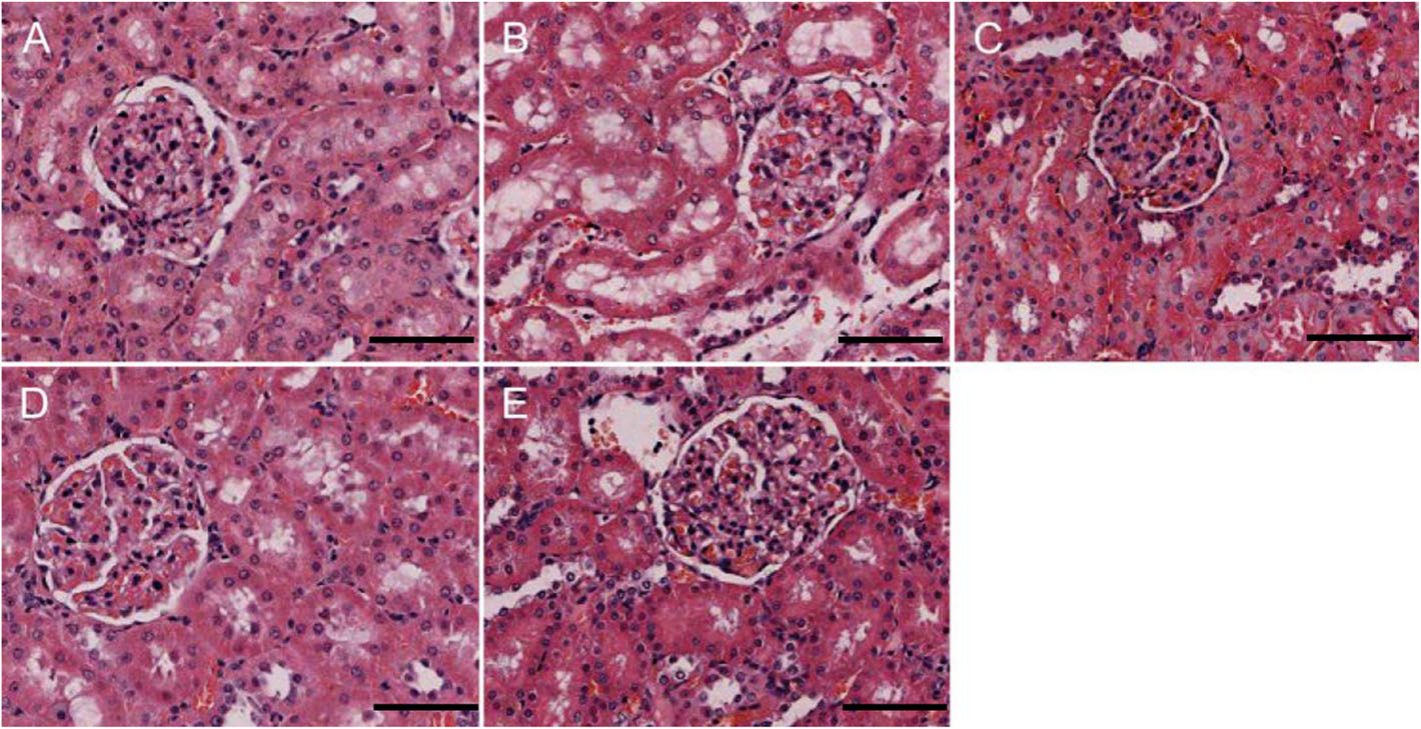
CIH causes renal damage. Changes in renal tissue morphology in each group were observed by HE staining. **a**: NC; **b**: 4w CIH; **c**: 6w CIH; **d**: 4w CIH + Ri; **e**: 6w CIH + Ri. Original magnification, × 200. Scale bars, 50 μm

**Fig. 2:**
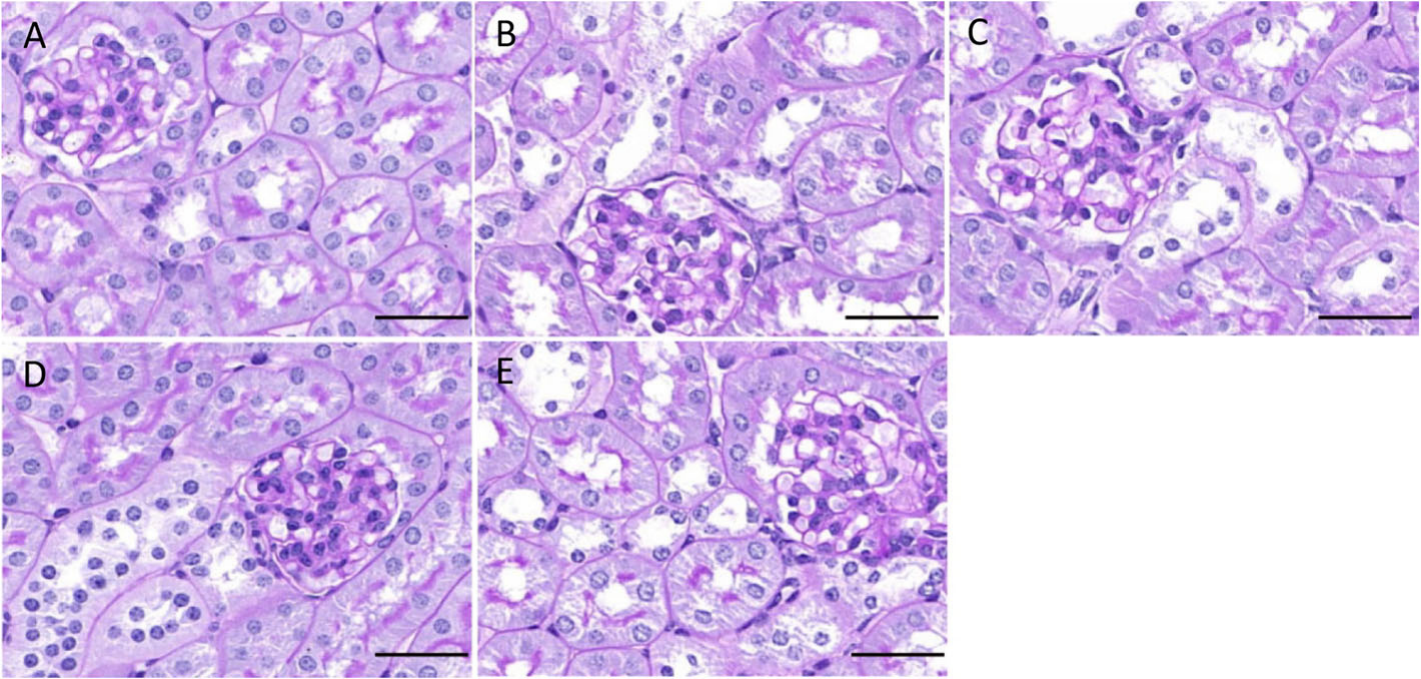
CIH causes renal damage. Changes in renal tissue morphology in each group were observed by PAS staining. **a**: NC; **b**: 4w CIH; **c**: 6w CIH; **d**: 4w CIH + Ri; **e**: 6w CIH + Ri. Original magnification, × 200. Scale bars, 50 μm

**Fig. 3:**
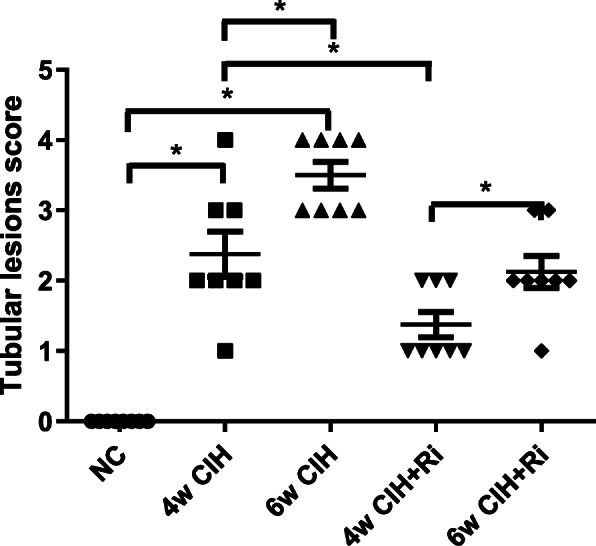
Tubular lesions score. Histopathological scoring was performed and data are expressed as means ± SEM, *n* = 8,^*^p < 0.05

Both HE and PAS staining showed that there were no abnormalities in glomerular and renal tubular epithelial cell morphology in the control group. The renal tubular epithelial cells in the 4w CIH group were moderately swollen, with sparse cytoplasm and tubule lumen was narrow. Some brush border of the tubules was lost while tubule dilatation, and the renal tubules were slightly damaged compared with those in the NC group (*p* < 0.05). There were no obvious abnormalities in the glomerulus. The tubular epithelial cells in the 6w CIH group were were future damaged (p < 0.05), the damage included extensive tubule dilatation, loss of brush border, swelling and exfoliation of tubular epithelial cell, tubular epithelial cell exfoliation and the tubule dilatation were aggravated. The glomerulus was slightly swollen, and the balloon gap was somewhat narrow. The renal tubular epithelial cells in the 4w CIH + Ri group were more neatly arranged and less swollen than those in the 4w CIH group. The tubular damage scores were decreased compared with those of the 4w CIH group (*p* < 0.05). The tubular epithelial cells in the 6w CIH+ Ri group were less swollen than the 6w CIH group, the loss of brush border and the tubule dilatation were reduced (*p* < 0.05). Meanwhile, the tubular damage scores were also decreased compared with those of the 6w CIH group (*p* < 0.05) (Figs. 1, 2, 3). The results indicated that the CIH groups had significantly higher damage scores than the NC group, Ri decreased the tubular damage scores.

### Morphological changes of mitochondria

**Fig. 4:**
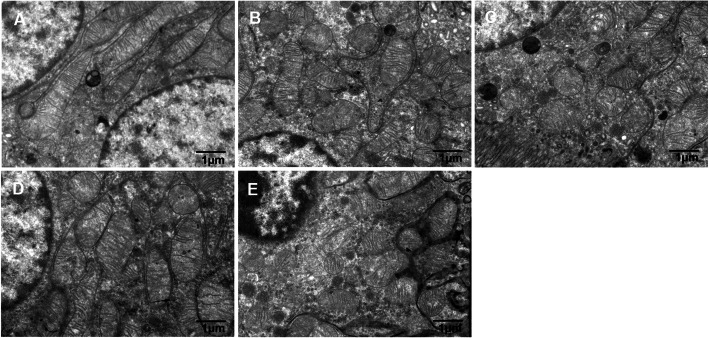
Mitochondrial ultrastructural changes in tubular epithelial cells. Representative pictures of Mitochondrial electron microscopy. **a**: NC; **b**: 4w CIH; **c**: 6w CIH; **d**: 4w CIH + Ri; **e**: 6w CIH + Ri. Original magnification, × 10,000. Scale bars, 1 μm

**Fig. 5:**
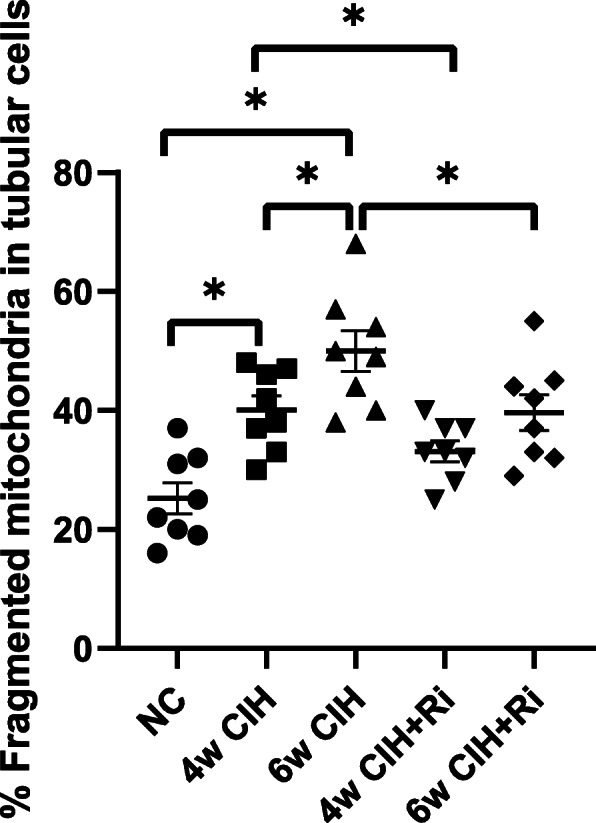
Quantitative analyses of the percentage of fragmented mitochondria in an average tubular cell in each group. Data are expressed as means ± SEM, *n* = 8,^*^p < 0.05

The ultrastructural changes of mitochondria in tubules from biopsied samples were assessed by electron microscopy. Extensive structure alteration in renal tubular epithelial cells were seen in CIH environment, mainly manifested in mitochondrial fragments. There were much elongated and cylindrical mitochondria in NC group, whereas the majority of mitochondria were shorter and round in CIH groups. Obviously, these changes in 6w CIH group were more serious than those in 4w CIH group (*P* < 0.05). After Ri treatment respectively, the mitochondrial fragments were reduced compared with those in 4w CIH group and 6w CIH group (*P* < 0.05) (Figs. 4, 5). These results suggested that Ri prevented the CIH-induced mitochondrial fragmentation in morphology.

### CB1R protein expression in rat renal tissue

**Fig. 6:**
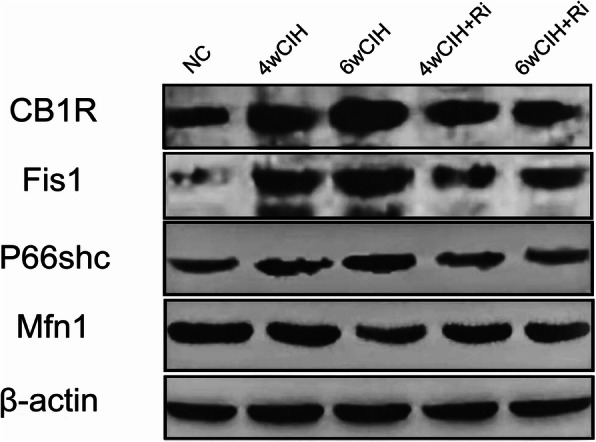
Representative western blotting images of CB1R, Fis1, p66shc and Mfn1 in renal tissue. Full-length blots are presented in Supplementary Figs. [1, 2, 3, 4, 5]

**Fig. 7:**
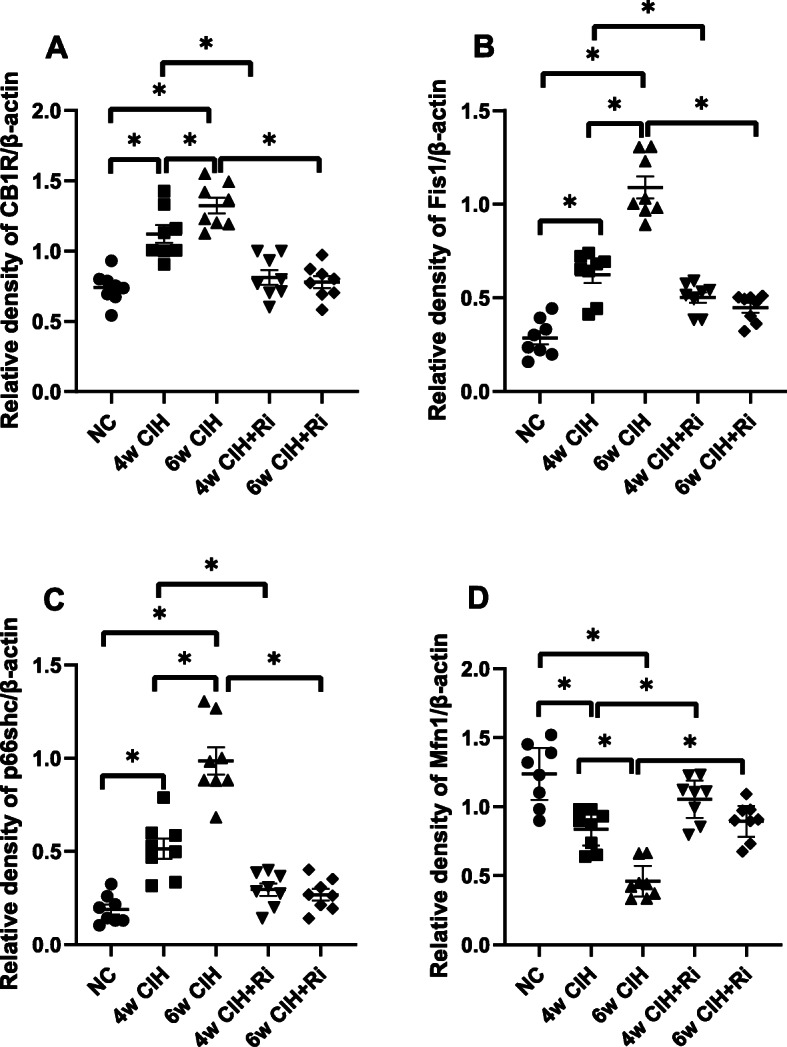
Western blot analyses of CB1R, Fis1, p66shc and Mfn1 levels and normalized to β-actin. Protein expression was measured with Image J. Data are expressed as means ± SEM, *n* = 8,^*^p < 0.001. **a**: Relative density of CB1R/β –actin; **b**: Relative density of Fis1/β–acti; **c**: Relative density of p66shc/β –actin; **d**: Relative density of Mfn1/β –actin

**Fig. 8:**
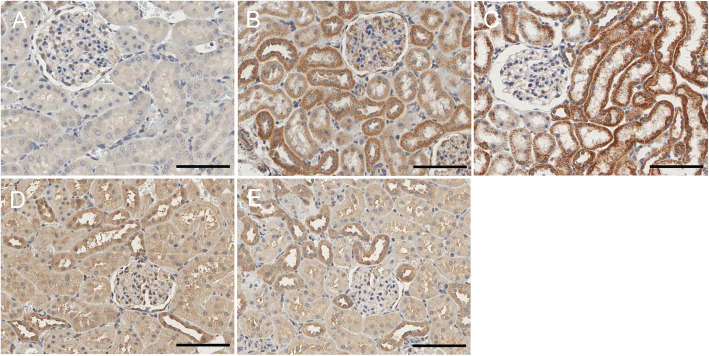
CIH increases renal CB1R levels, which are attenuated by rimonabant. CB1R immunohistochemistry in renal tissue in each group. **a**: NC; **b**: 4w CIH; **c**: 6w CIH; **d**: 4w CIH + Ri; **e**: 6w CIH + Ri. Original magnification, × 200. Scale bars, 50 μm

The results of immunohistochemistry and Western blotting demonstrated that CB1R expression in the renal tissue of 4 and 6w CIH groups was increased (*p* < 0.05), and the renal tubules in 4 and 6w CIH groups were damaged. Compared with 4w CIH, the CB1R in renal tissue of rats exposed to CIH 6w was further increased (*p* < 0.05) and the renal injury was more serious. After treatment with the CB1R antagonist Ri, the level of CB1R was significantly lower than that of CIH groups (*p* < 0.05), and the injury of renal tissue was also alleviated (Figs. 6, 7, 8, and 12a, a). The results showed that Ri could reduce the increase of CB1R induced by CIH, and thus alleviate the kidney injury.

### Fis1/p66Shc/Mfn1 protein expression in rat renal tissue

**Fig. 9:**
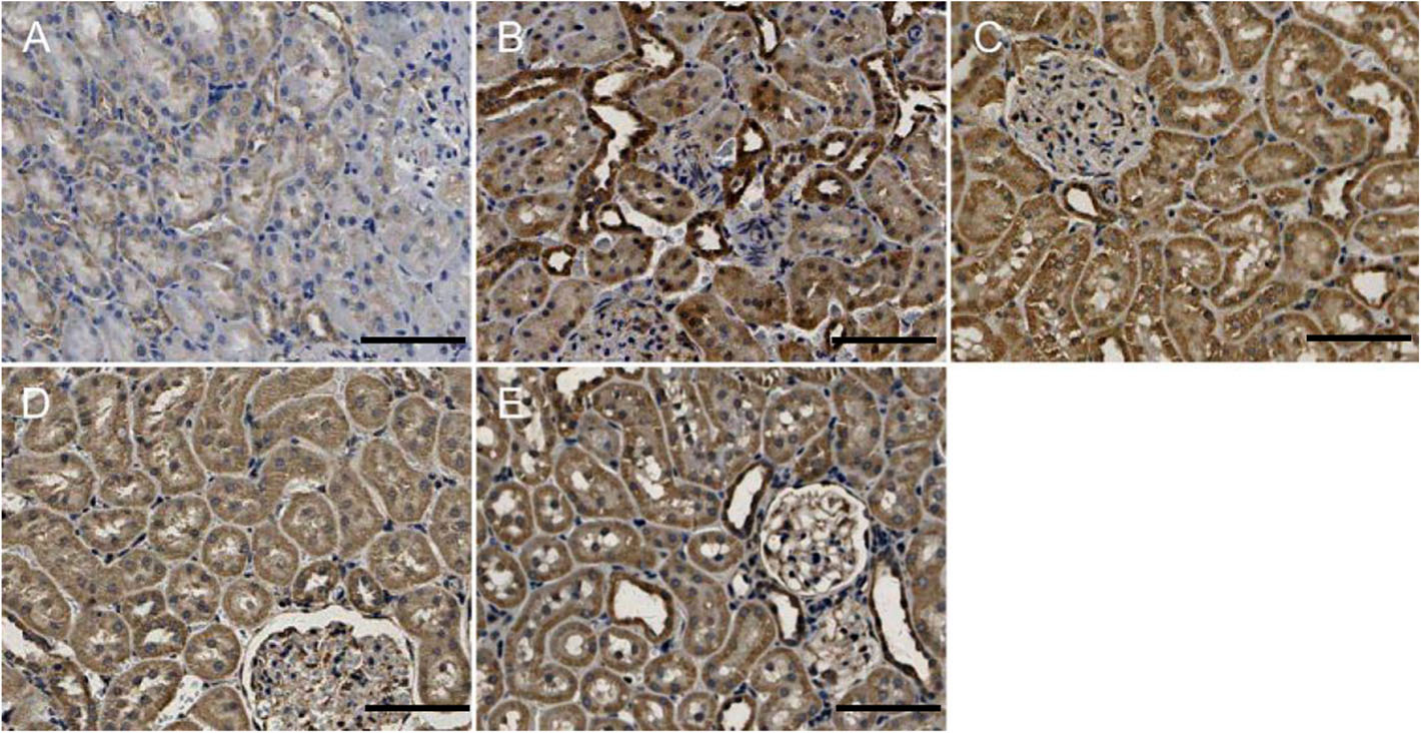
Fis1 protein expression in rat renal tissue. Immunohistochemical labelling of Fis1 in renal tissue in each group. **a** NC; **b**: 4w CIH; **c**: 6w CIH; **d**: 4w CIH + Ri; **e**: 6w CIH + Ri. Original magnification, × 200. Scale bars, 50 μm

**Fig. 10:**
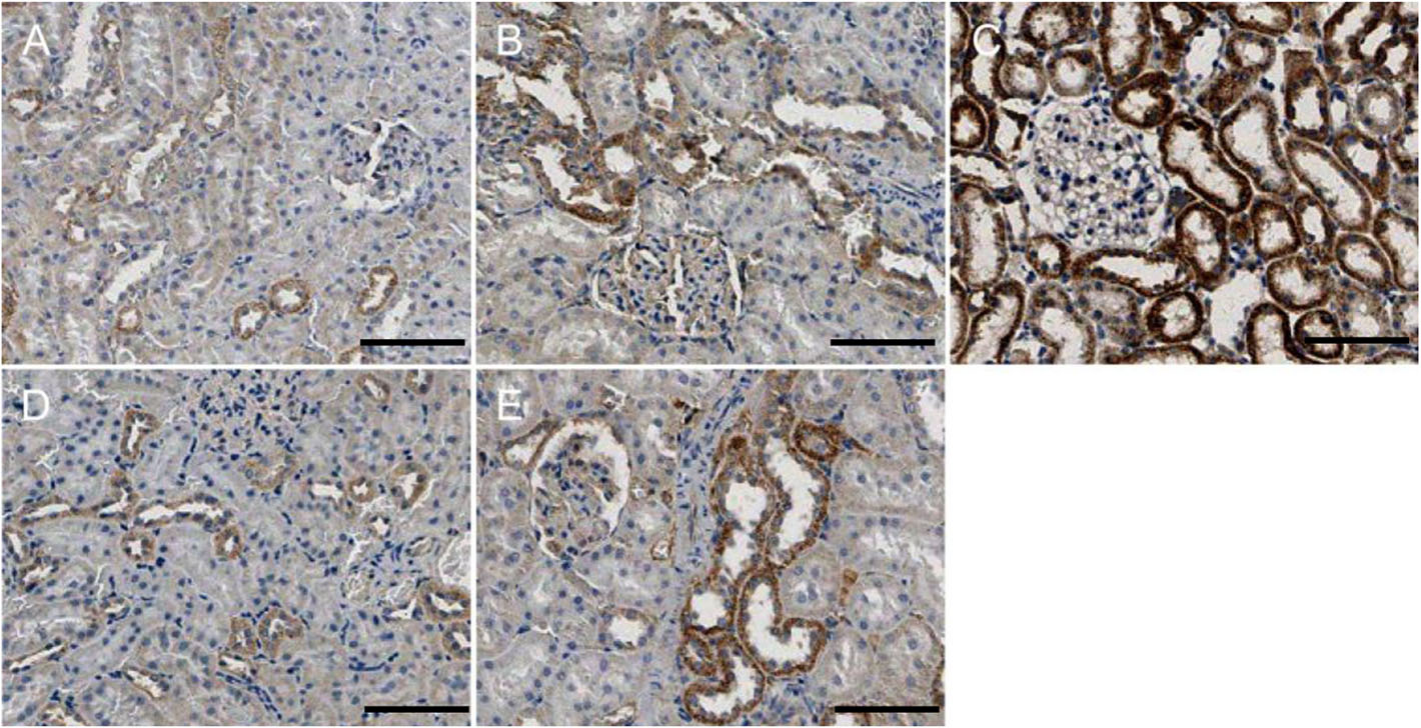
P66shc protein expression in rat renal tissue. Immunohistochemical labelling of p66shc in renal tissue in each group. **a**: NC; **b**: 4w CIH; **c**: 6w CIH; **d**: 4w CIH + Ri; **e**: 6w CIH + Ri. Original magnification, × 200. Scale bars, 50 μm

Western blotting analyses and immunohistochemistry showed that Fis1 and p66Shc were expressed in the glomeruli and tubules of each group. The cytoplasm was yellowish or brown under the light microscope. In the control group, there was little expression in the glomeruli and tubules. Fis1 and p66Shc protein were significantly increased in CIH group renal tubules, with the highest expression in the 6w CIH group. Compared with the 4w CIH group, Fis1 and p66Shc protein expression in the 4w CIH + Ri intervention group were significantly decreased (*p* < 0.05) and when compared with the 6w CIH group, Fis1 and p66Shc protein expression were significantly decreased (*p* < 0.05) in the 6w CIH + Ri group (Figs. 6, 7, 9, 10, 12b, c, b and c).

**Fig. 11:**
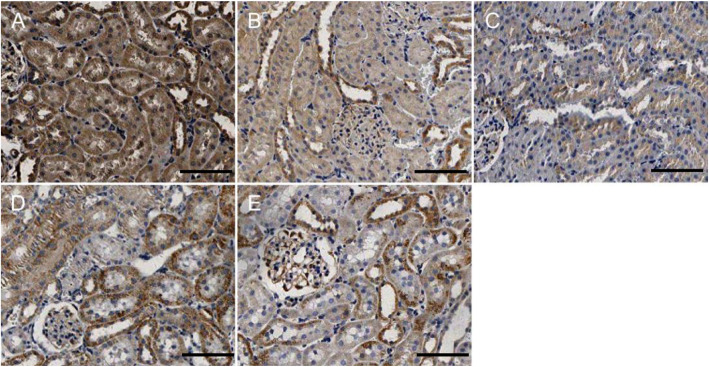
Mfn1 protein expression in rat renal tissue. Immunohistochemical labelling of Mfn1 in renal tissue in each group. **a**: NC; **b**: 4w CIH; **c**: 6w CIH; **d**: 4w CIH + Ri; **e**: 6w CIH + Ri. Original magnification, × 200. Scale bars, 50 μm

**Fig. 12:**
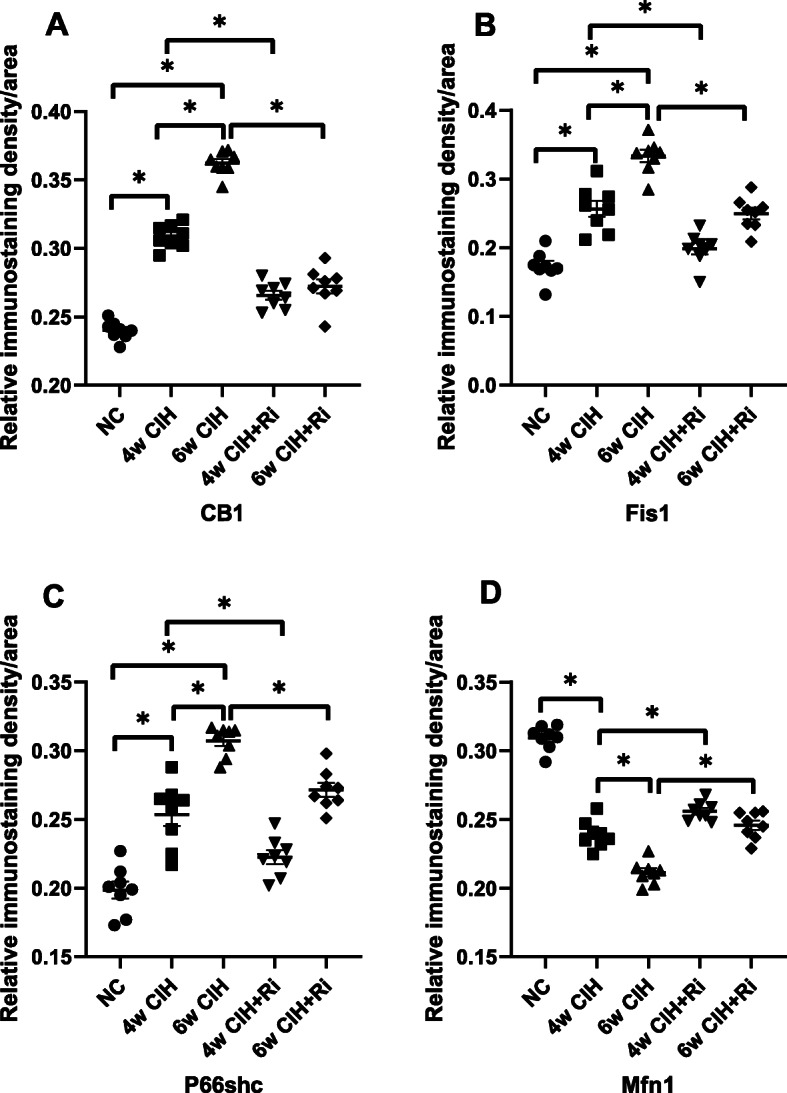
Quantitative analyses of CB1R, Fis1, p66shc and Mfn1 using immunohistochemistry. Data are expressed as means ± SEM, *n* = 8,^*^p < 0.05. **a**: Relative immunostatining density of CB1R/aera; **b**: Relative immunostaining density of Fis1/area; **c**: Relative immunostaining density of P66shc/area; **d**: Relative immunostatining density of Mfn1/area

Western blotting analyses and immunohistochemistry also showed that Mfn1 was expressed in the glomeruli and tubules of each group of rats, and the cytoplasm of each group was light yellow or brown. In the control group, there was high Mfn1 expression in the glomeruli and tubules. Mfn1 protein expression in renal tubules was significantly decreased (*p* < 0.05) in the 4 or 6w CIH group, with the lowest expression in the 6w CIH group. Compared with the 4w CIH group, Mfn1 protein expression in the 4w CIH + Ri group was significantly increased(*p* < 0.05) and when compared with the 6w CIH group, Mfn1 protein expression in the 6w CIH + Ri group was significantly increased(*p* < 0.05) (Figs. 6, 7, 11 and 12d, d).

These results suggest that Ri not only alleviated the morphological fragment in kidney which induced by CIH, but also changed the expression of mitochondrial dynamics proteins Fis1, p66Shc and Mfn1 in CIH model, ultimately reduced CIH-induced renal injury.

### Correlation comparison

Pearson correlation analysis showed a positive correlation between CB1R and Fis1 expression (R^2^ = 0.797, *p* < 0.01), and CB1R and p66shc (R^2^ = 0.659, *p* < 0.01). Furthermore, CB1R and Mfn1 were negatively correlated (R^2^ = − 0.737, *p* < 0.01; see Table 1).

**Table 1:** Correlation between CB1R expression and Fis1, p66shc and Mfn1 levels

Index	R^2^-value	*p*-value
Fis1	0.797	*p* < 0.01
p66shc	0.659	*p* < 0.01
Mfn1	− 0.737	*p* < 0.01

## Discussion

CIH is the foremost pathophysiological change of OSA, and contributes to continued renal function deterioration. We used a CIH rat model to simulate the OSA pathophysiologic processes, and observed that CIH induced serious renal structure damage in the CIH group. This damage included extensive tubule dilatation, loss of brush border, swelling and exfoliation of tubular epithelial cell. The degree of abnormal changes in renal tissue is associated to CIH in a time-dependant manner. Moreover, Ri notably alleviated these morphological changes, indicating that renal injury was significantly hampered in the CIH + Ri groups. Therefore, we conclude that pathological changes from OSA in CIH may cause pathological changes in renal tissue.

EC disorders were detected in OSA patients in early clinical studies [32, 33], which include CB1R overexpression. Furthermore, many enzymes that synthesize ECs were increased in these patients. CB1R is often used to detect changes in EC system function. In this study, CB1R is expressed in renal tissue. Compared with normal tissue, CB1R expression was increased in the CIH groups in a time-dependent manner; however, Ri reduced CB1R expression in both CIH groups. There were no obvious changes among NC groups. Thus, we conclude that CIH increases CB1R expression in renal tissues, and that the expression level is related to hypoxia duration and intervention.

Recently, mitochondrial dynamics in various kidney diseases have been extensively investigated. Current reports mainly focus on diabetic kidney disease, renal ischaemia-reperfusion injury, and drug-induced or heavy-metal nephropathy. Studies have shown that the expression of mitochondrial dynamic proteins, Drp1 and Fis1, are upregulated, while Mfn1 expression is down-regulated in early-stage renal disease. These alterations induce changes in mitochondrial morphology and dynamics, cause mitochondrial fragmentation, promote mitochondrial dysfunction, and lead to oxidative damage and apoptosis [34–39]. Interestingly, p66Shc is a master regulator of mitochondrial ROS, apoptosis, and lifespan in mammals, and is involved in several diseases, particularly aging and metabolic disorders [40]. Following the induction of stress, p66Shc is activated and phosphorylated in the cytosol, inducing translocation to the mitochondrial inter-membrane space, where it binds and oxidizes cytochrome C to generate excessive ROS (H_2_O_2_) that leads to apoptosis [41, 42]. These results link p66Shc to mitochondrial dynamics and apoptosis in tubular cells in diabetic nephropathy, and identify a novel mechanism underlying the redox-regulating and pro-apoptotic effects of p66Shc. In this study, we observed that p66Shc, Fis1, and Mfnl are expressed in renal tubular epithelial cells. p66Shc and Fis1 expression was significantly increased in the CIH group, while Mfnl expression was significantly decreased. Excitingly, the electron microscope also showed that mitochondria of renal tubular epithelial cells were widely fragmented in CIH environment, the degree of mitochondrial fragmentation depended on the duration of CIH. The results indicate that abnormal mitochondrial dynamics may participate in CIH-induced kidney disease progression, and the expression of p66Shc, Fis1 and Mfn1 is closely related to disease severity. Besides, we observed that p66Shc, Fis1, and Mfnl expression were closely related to CB1R expression. Further, Ri increased Mfnl expression, decreased Fis1 and p66Shc expression, alleviated the over division of mitochondria, and ameliorated renal tissue injury. These results suggest that CB1R activation leads to mitochondrial fragmentation, and that Ri reduces CIH-induced renal damage by inhibiting altered mitochondrial dynamics.

In conclusion, we demonstrate that CIH caused by OSA could trigger EC system disorders, resulting in renal injury. Further, our results indicate that CIH increases CB1R expression, which increases p66Shc and induces mitochondrial dynamic alterations by disrupting fission-fusion machinery. This disruption can result in the loss of mitochondrial membrane potential, cytochrome C release, and consequent oxidative stress and apoptosis [22]. After using CB1R antagonists, CB1R expression decreases, indicating restored mitochondrial dynamics, which further improved the renal damage. These results indicate that CB1R plays a role in renal injury caused by CIH, and CB1R inhibition decreases the risk of CIH patients from developing EC system disorders. Thus, the EC system could be a therapeutic target to ameliorate renal injury caused by OSA. However, neither did we study the changes in CB1R and renal tissue under different hypoxia conditions, nor any dose-dependent protective effects of Ri on CIH-induced renal injury in our animal study. Hence, these components warrant further investigated in future studies.

## Supplementary Information

**Additional file 1: Figure S1.** Representative western blotting images of CB1R in renal tissue. **Figure S2.** Representative western blotting images of Fis1 in renal tissue. **Figure S3.** Representative western blotting images of p66shc in renal tissue. **Figure S4.** Representative western blotting images of Mfn1 in renal tissue. **Figure S5.** Representative western blotting images of β- actin.

## Data Availability

The datasets used and /or analyzed in the current study are available from the corresponding author on reasonable request.

## Abbreviations

CB1R: Cannabinoid receptor 1
CIH: Chronic intermittent hypoxia
CKD: Chronic kidney disease
EC: Endocannabinoid
FiO_2_: Fraction of inspired oxygen
NC: Normal control
OSA: Obstructive sleep apnoea
Ri: Rimonabant
ROS: Reactive oxygen species
