# Supplementary material for: CB1 receptor antagonist rimonabant protects against chronic intermittent hypoxia-induced renal injury in rats
Source: BMC Nephrol. 2021 Apr 26;22:153. doi: 10.1186/s12882-021-02362-6 (PMC8077827; doi:10.1186/s12882-021-02362-6)
Supplement: Supplementary file 1 — Additional file 1: Figure S1. Representative western blotting images of CB1R in renal tissue. Figure S2. Representative western blotting images of Fis1 in renal tissue. Figure S3. Representative western blotting images of p66shc in renal tissue. Figure S4. Representative western blotting images of Mfn1 in renal tissue. Figure S5. Representative western blotting images of β- actin. [file 12882_2021_2362_MOESM1_ESM.docx]

**Supplemental figures of full-length blots**

Li Zhao, Tao Liu, Zhan-jun Dou, Mei-ting Wang, Zi-xuan Hu, and Bei Wang*

Li Zhao MD, Tao Liu M.M, Zhan-jun Dou MD, Mei-ting Wang M.M, Zi-xuan Hu M.M, Shanxi Medical University, No. 56, Xijian South Road, Taiyuan 030001, Shanxi, P.R. China

*Correspondence to:

Dr Bei Wang MD, The Second Hospital of Shanxi Medical University, No. 382, Wuyi Road, Taiyuan 030001, Shanxi Province, P.R. China

Email: myemaila@qq.com

Tel: +86-13623451511


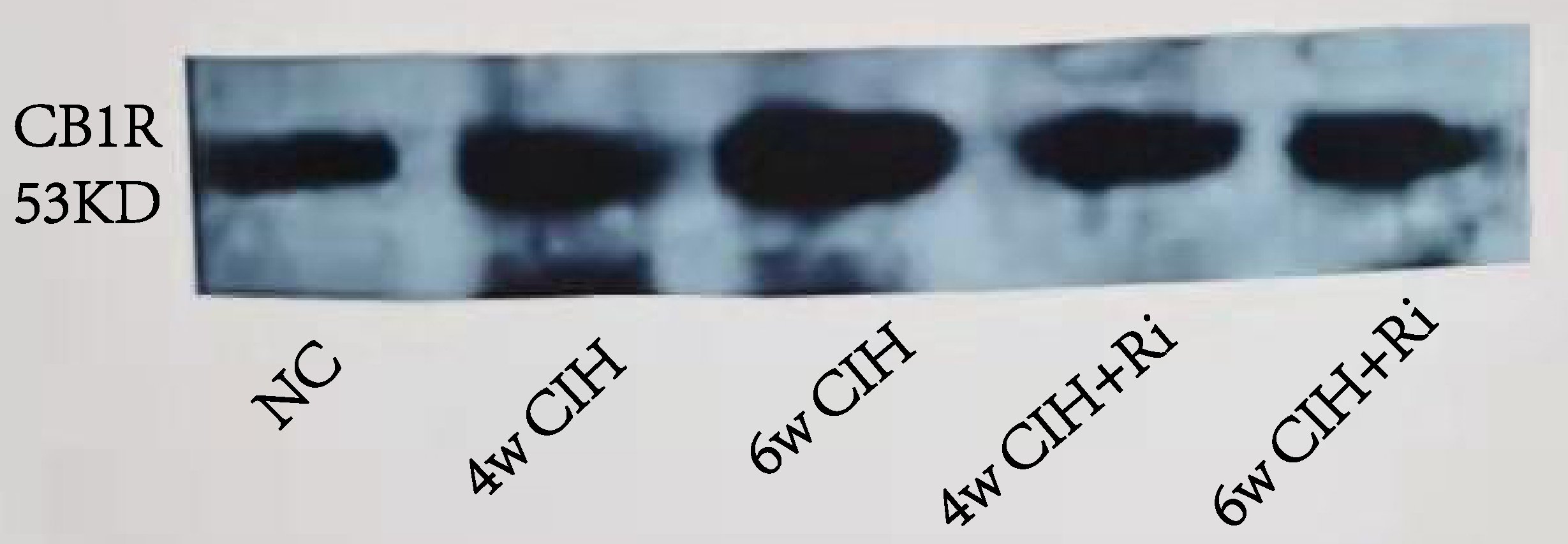


**Figure S1.** Representative western blotting images of CB1R in renal tissue.


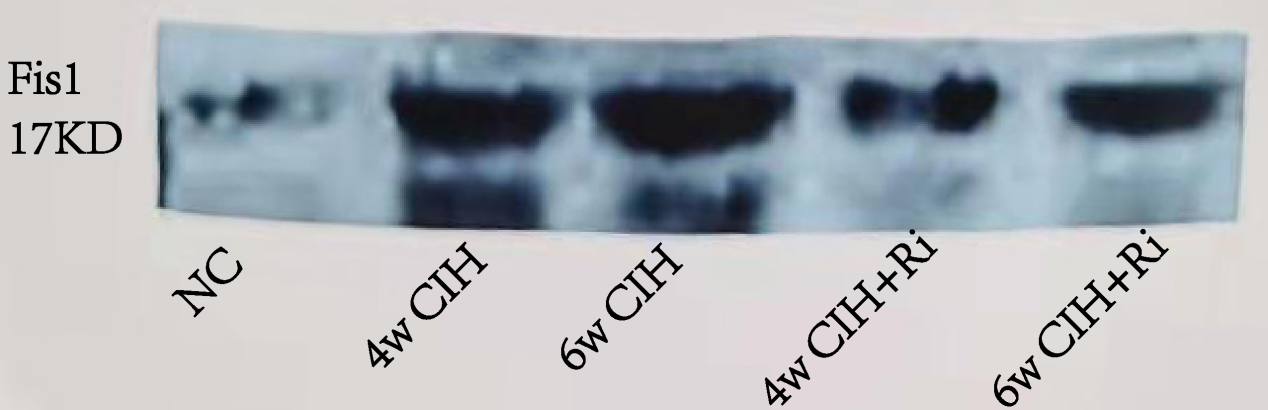


**Figure S2.** Representative western blotting images of Fis1 in renal tissue.


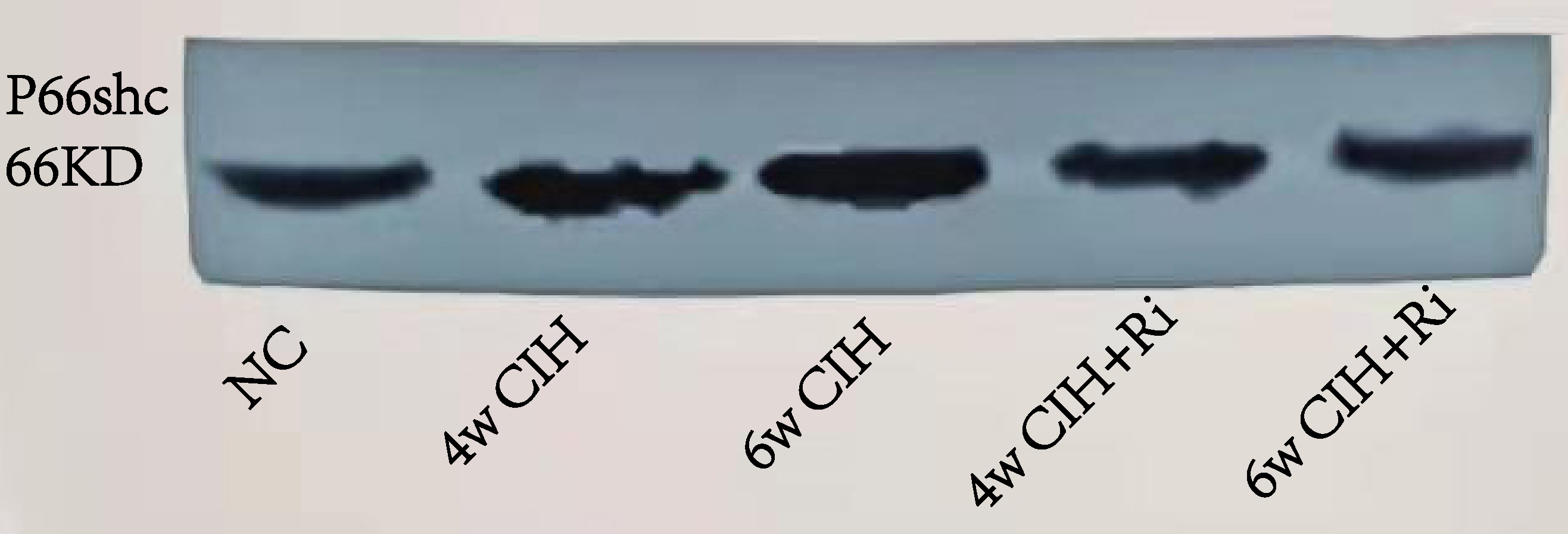


**Figure S3.** Representative western blotting images of p66shc in renal tissue.


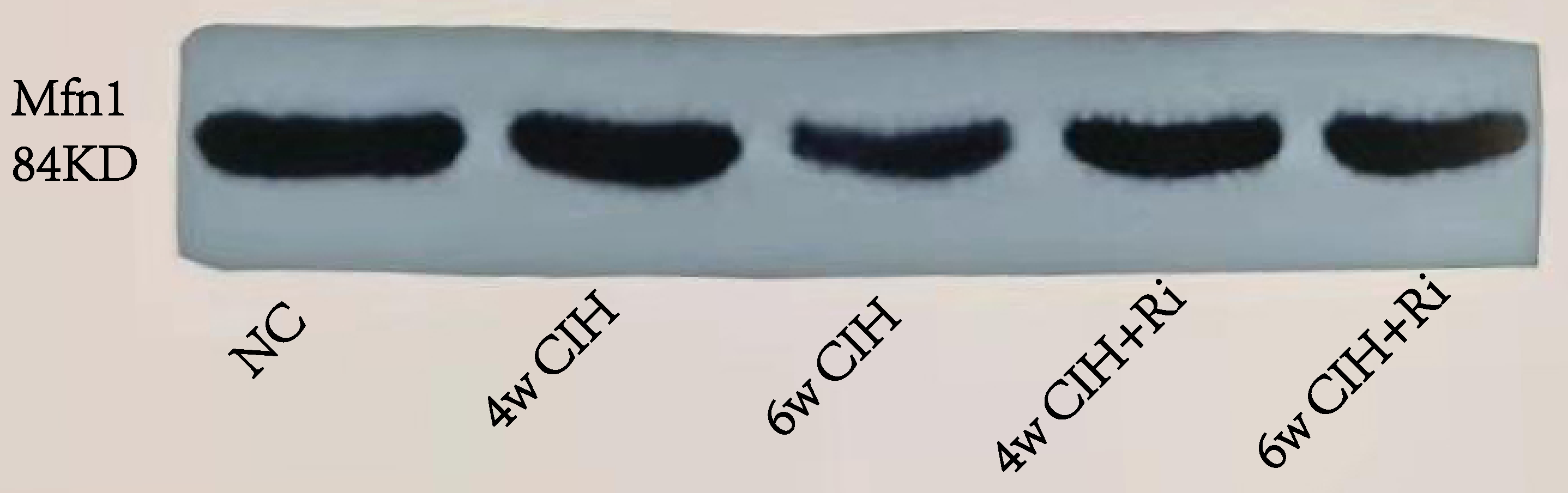


**Figure S4.** Representative western blotting images of Mfn1 in renal tissue.


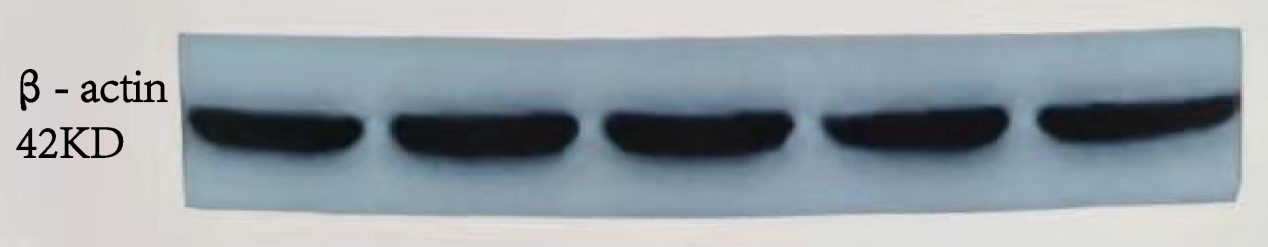


**Figure S5.** Representative western blotting images of β- actin.
